# Evaluation of the accuracy of Cone Beam Computerized Tomography (CBCT): medical imaging technology in head and neck reconstruction

**DOI:** 10.1186/1916-0216-42-25

**Published:** 2013-03-21

**Authors:** Heather Logan, Johan Wolfaardt, Pierre Boulanger, Bill Hodgetts, Hadi Seikaly

**Affiliations:** 1 Institute for Reconstructive Sciences in Medicine, Misericordia Community Hospital Edmonton, 1W-02, 16940-87 Avenue, Alberta, T5R 4H5, Canada; 2 Department of Computing Science, University of Alberta, Athabasca Hall, Room 411, Edmonton, Alberta, T6G 2E8, Canada; 3 Speech Pathology and Audiology, University of Alberta, 2-16, Corbett Hall Edmonton; 116 St. and 85 Ave, Edmonton, AB, T6G 2 G4, Canada; 4 Department of Otolaryngology-Head & Neck Surgery, 8440 - 112th Street, 1E4.34 WMC, Edmonton, AB T6G 2B7, Canada

## Abstract

**Background:**

With the introduction, development and commercialization of Cone Beam Computerized Tomography (CBCT) technologies in the field of head and neck reconstruction, clinicians now have increased access to the technology. Given the growth of this new user group, there is an increasing concern regarding proper use, understanding, quality and patient safety.

**Methods:**

The present study was carried out to evaluate data acquisition of CBCT medical imaging technology and the accuracy of the scanning at three different machine warming times. The study also compared the accuracy of CBCT at 0.2 mm slice thickness and Computerized Tomography (CT) at 1 mm slice thickness. A control model was CT scanned at five random intervals, at 1 mm slice thickness and CBCT scanned at specialized intervals, at 0.2 mm slice thickness. The data was then converted and imported into a software program where a digital registration procedure was used to compare the average deviations of the scanned models to the control.

**Results:**

The study found that there was no statistically significant difference amongst the three CBCT machine warming times. There was a statistically significant difference between CT scanning with 1 mm slice thickness and CBCT scanning with 0.2 mm slice thickness.

**Conclusions:**

The accuracy of the i-CAT CBCT scans used in the present study with a parameter at voxel size 0.2, will remain consistent and reliable at any warming stage. Also the difference between the CBCT i-CAT scans and the CT scans was not clinically significant based on suggested requirements of clinicians in head and neck reconstruction.

## Background

Three-dimensional (3D) information has become an important tool in assisting diagnosis and surgical planning in the oral and maxillofacial field. Many professionals in the field have been limited in the use of medical imaging technologies that produce 3D images and models due to availability, restricted access and radiation dose considerations [1]. With the introduction, development and commercialization of CBCT technologies, clinicians now have increased access to the technology. Given the growth of this new user group, there is an increasing concern regarding proper use, adequate understanding, optimal quality and patient safety [1]. It is important for users of this technology to be well informed and trained in all the technical implications of creating a proper and accurate 3D surface model. The quality of the CBCT scan depends on the scanner type, the scanning parameters and the reconstruction settings [2]. There is a significant volume of information in the literature that describes the applications of CBCT technologies [3-7], but few studies examine the important technical protocols that need to be followed.

The present study is intended to contribute to the growing field of CBCT imaging technologies, contribute to the improvement of scanning techniques and protocols and bring knowledge to clinical users. Its general purpose is to examine the accuracy of CBCT and CT scanning technologies using 3D surface model reconstructions. There are two specific objectives. The first is to assess whether there is a difference in accuracy between CBCT scanning at three difference machine warming stages. The warming period of the CBCT was explored in order to understand whether sensitivity and precision of the sensor in the CBCT is affected by thermal distortion. The second is to assess whether there is a difference in accuracy between CT scanning and CBCT scanning at the three warming stages.

## Methods

**Figure 1:**
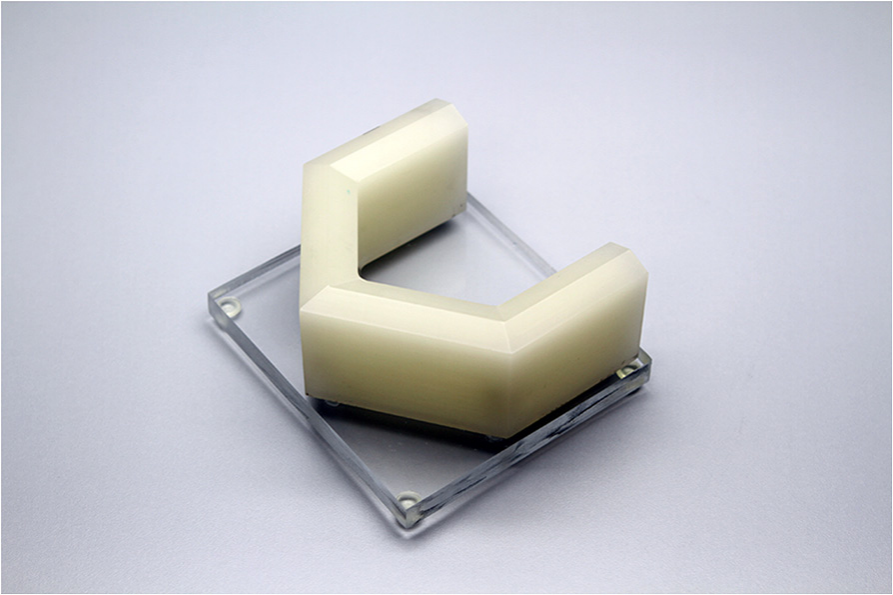
Model and platform design for CBCT and CT scanning.

A computer aided design (CAD) model was designed in the software program Rhinoceros 4.0 (McNeel North America, Seattle, WA, USA). The model was designed to represent similar dimensions and contours of a standardized mandible chosen by the researcher but with flat planes (Figure 1). The model was then milled out of an acrylic resin (ProBase Cold, Ivoclar Vivadent AG Technical, Schaan Liechtenstein) block mixed with barium sulfate (E-Z-HD, Anjou Quebec, Canada) by Southern Implants, South Africa. The barium sulfate (5% based on the total weight) was added to the acrylic resin to ensure visibility of the material as a digital 3D volume. A platform was designed to hold the model in place and to slightly elevate the model to allow for proper segmentation after 3D reconstruction of the CT and CBCT scans.

The milled model was then scanned using the Kreon laser scanner (Kreon Technologies,Limoge, France) mounted on a FARO Arm Laser Scanner (FARO Technologies Inc. Lake Mary, FL) in order to produce a representation of the model that was highly accurate. The FARO arm is a mechanical arm with a precision of 15 microns on which the Kreon 3D scanner is mounted that was precise to 40 microns. The combined precision of the scanner and the arm was 55 microns. The laser scan surface model produced by the 3D scanner was the control model for comparison.

A protocol was formulated to ensure that every CBCT scan and CT scan was completed with precision and consistency. The CBCT scanning protocol included a calibration technique that involved conducting a weekly Panel Calibration as outlined in the manufacturer’s recommended protocol for the system (i-Cat 17–19, Imaging Sciences International LLC, Hatfield, PA, USA). Both the CBCT and the CT protocols included steps for orientation verification and technical parameters (Tables 1 and 2) to ensure optimal scanning.

**Table 1:** CBCT Parameters (i-Cat 17–19, Imaging Sciences International LLC, Hatfield, PA USA) used for all scanning

	
Tube Voltage (kilovolt kV)	120 kVp
Tube Current (Milli-ampere mA)	5 mA
Tube Current x Exposure Time	37.07 mAs
(Milli-ampere x seconds mAs)	
Acquisition Time	26.9 seconds
Diameter	16 cm
Height	8 cm
Voxel	0.2 voxel

**Table 2:** CT Parameters (Toshiba Medical Systems, Aquilion 64) used for all scanning

Gantry tilt	0 degree
Kernel setting	Soft
Bone window	High Resolution
Slice increment	1 mm slices
Anatomical plane data required	Axial helical
Occlusal plane position	Perpendicular to the gantry

Each CBCT scan was performed at the Institute for Reconstructive Sciences in Medicine (iRSM), Edmonton, Alberta, Canada. A voxel size of 0.2 mm was chosen in order to obtain the highest resolution for 3D volume reconstruction. All CT scans were completed by the lead CT technologist in the Department of Diagnostic Imaging (DI) at the Misericordia Community Hospital in Edmonton, Alberta, Canada. The CT protocol, which included 1 mm slices (using 1 mm overlap), was intended to mimic the guidelines of medical modeling used for patients of iRSM.

**Figure 2:**
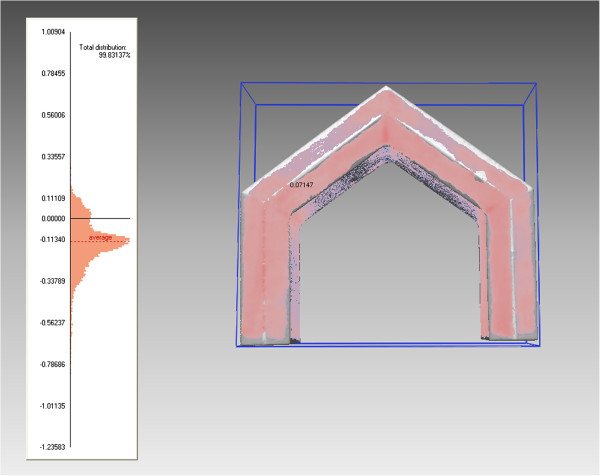
Representation of a color-map inspection produced in Rapidform 2006.

**Figure 3:**
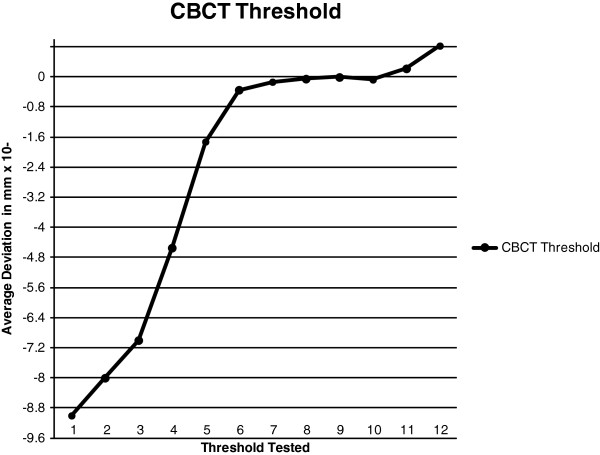
CBCT optimal threshold testing.

**Figure 4:**
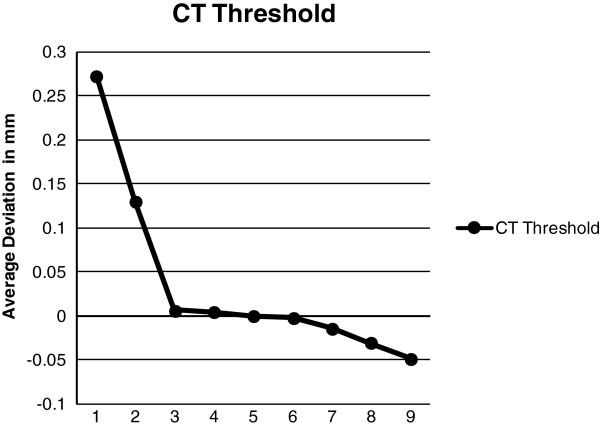
CT optimal threshold testing.

In order to understand whether the length of time the CBCT scanner has been turned on affects the accuracy of the scan, the CBCT scans were recorded at three separate times within one day. The first scan was completed with the machine cold, i.e. as soon as the iCAT was turned on. The second scan was done one hour after the machine was turned on and the third scan was four hours after the machine was turned on. The warming period of the CBCT was explored in order to understand whether sensitivity and precision of the sensor in the CBCT is affected by thermal distortion. This procedure was repeated for a total of six days. The CT scans were done whenever possible due to limited access to the scanning equipment. A total of five CT scans were completed at random times. Once the scans were completed, each scan was exported in a Digital Imaging and Communications in Medicine (DICOM) file format and was then brought into the software program InVivoDental 5.0 Anatomy Imaging Software (Anatomage Inc. San Jose, CA. USA) in order to construct a 3D surface model. The optimal threshold for the material of the control model and the particular scan was then searched for by testing several different thresholding levels. An optimal threshold was searched for in order to obtain an accurate representation of the scanned model, not only to show the difference between thresholding for CBCT and CT but also because the control model was made of acrylic resin and barium sulphate, and hence is a material that does not have a specified Hounsfield Units. The researcher estimated a number for the thresholding level, converted the DICOM file to a stereolithography (.stl) CAD file, imported the model into Rapidform 2006 computer software (Rapidform Inc. Seoul, South Korea) and followed the automatic registration procedure to register the tested threshold model to the control model. After the initial alignment, a global registration was done in order to precisely match the position of all the selected shells at the same time using the overlapped region that was automatically found by the software. Once the global registration was complete, the registration was evaluated using the shell/shell deviation calculation. For each point in the point cloud of each model, the deviation operation found the 3D distance to the respective closest point in the surface list. The software calculated and created a color plot that visually and textually represented maximum/minimum and average, positive and negative deviations from actual to the theoretical virtual images. Every color represented a dimension in millimeters indicating the difference between the two virtual images (Figure 2). The optimal threshold was chosen based on the point-to-point average deviation that was closest to zero. This was completed separately for the CBCT scans (Figure 3, Table 3) and CT scans (Figure 4, Table 4).

**Table 3:** **Threshold levels represented in Figure **3

**Number represented**	**Threshold tested**
1	−183
2	−172
3	−160
4	−100
5	−50
6	−20
7	−16
8	−15
9	−14*
10	−13
11	−10
12	0

* = Optimal threshold chose.

**Table 4:** **Threshold levels represented in Figure **5

**Number represented**	**Threshold tested**
1	−17
2	−100
3	−170
4	−171
5	−172*
6	−173
7	−180
8	−190
9	−200

* = Optimal threshold chosen.

Once the optimal threshold was found, a consistent threshold was kept for all CBCT scans and for all CT scans. The resulting 3D models were then exported as stl files.

## Results

### Accuracy of CBCT at three machine warming stages

**Figure 5:**
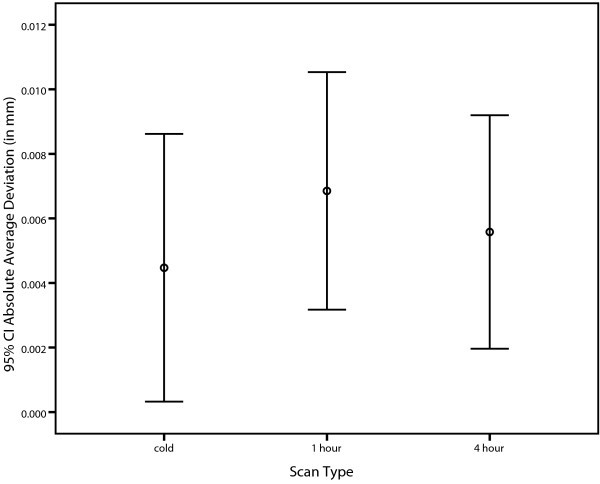
Error bar graph showing the absolute average deviation measurements (in mm) for CBCT scans cold, 1 hour and 4 hour.

As shown in Figure 5, all bars overlap which is indicative that is there is no difference between the three machine warming stages. As shown in Table 5, the mean values of CBCT cold and 4 hour appeared to be very similar while CBCT 1 hour appeared to be slightly higher. The one-way ANOVA revealed that there is no significant difference between CBCT (cold), CBCT (1 hour) and CBCT (4 hour) (F(2,15) = .341, p = .717).

**Table 5:** Descriptive statistics for absolute average deviation (in mm) from the target control model

**Scan type**	**Minimum**	**Maximum**	**Mean**	**SD**	**Skewness**	**Kurtosis**
**CBCT cold**	0.00003	0.01324	0.00593	0.00467	0.616	0.28
**CBCT 1 hour**	0.00323	0.01466	0.00815	0.00415	0.62000	−0.31500
**CBCT 4 hour**	0.00268	0.01839	0.00772	0.00584	1.54800	2.11700
**CT**	0.05146	0.06430	0.05614	0.00513	1.15700	1.29200

### Accuracy of CT and CBCT at three machine warming stages

**Figure 6:**
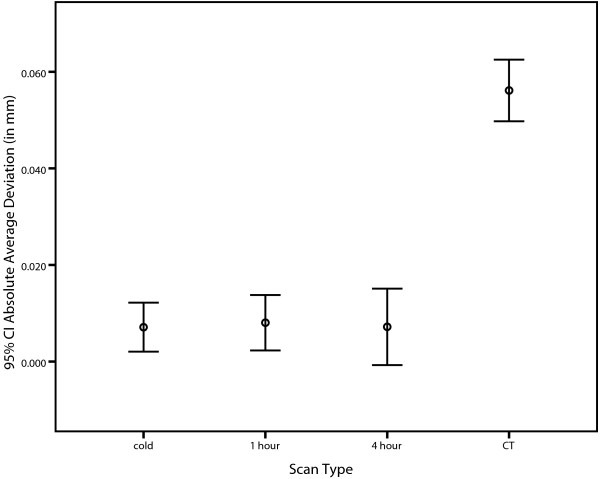
Error bar graph showing the absolute average deviation measurements (in mm) for CBCT (0.2 mm slice thickness) scanning cold, 1 hour and 4 hour and CT scanning (1mm slice thickness).

As shown in Figure 6, the error bars show that the mean values of CBCT cold, 1 hour and 4 hour were very similar while CT scan type was much greater. This is also shown in the descriptive statistics in Table 5. There was a statistically significant effect of scan type (F(3,19) = 125.979, p < .0005, partial η^2^ = .952). Employing the Bonferroni post-hoc test, significant differences were found between the CT (1 mm slice thickness) and the CBCT (cold 0.2 mm slice thickness) (p < .0005), between CT (1 mm slice thickness) and the CBCT (1 hour 0.2 mm slice thickness) (p < .0005) and CT (1 mm slices) and the CBCT (4 hour 0.2 mm slice thickness) (p < .0005).

## Discussion

The present study was faced with a number of unanticipated issues that added complexity. Thresholding became an important technical challenge in the process of data acquisition and conversion in the study. The researcher was left to estimate the optimal threshold level and this affected the shape and accuracy of the 3D surface model reconstruction. Throughout the process of the study, the topic of thresholding was considered within the scope of the research project and discussed with an expert in the field of computer science as well as with technical support employees of the i-CAT imaging technology and software. Interestingly, the present study revealed ambiguity, inconsistency and a lack of understanding among several sources on this topic. It was decided to maintain consistency wherever possible in the thresholding procedure. Although a consistent threshold level was used in the present study due to limitations in the scope of the research project, it is important to note that every scan completed by the CBCT will have a different optimal threshold. For example, it was found that the optimal threshold for the CBCT scans completed on day five were all different. The optimal threshold for CBCT scan cold was −8, 1 hour was −20 and 4 hour was −21.

In general, the present study revealed important findings that contribute to the growing body of knowledge and technical understanding in the area of CBCT and medical imaging technology. In terms of its first objective, evaluating the accuracy of CBCT at three machine warming stages, the study showed no statistically significant differences among the periods for the machine tested. It is important to consider that the overall averages of the average deviation of all the warming periods were smaller than 0.005 of a mm (that is one 200^th^ of a mm). This number possibly could have been smaller with optimal thresholding levels for each single scan. Given that the resulting averages were of very small magnitude, the clinical significance of the mean differences is considered to be of no concern. It was felt that clinicians can be confident that the accuracy of the i-CAT CBCT scans used in the present study with a parameter at voxel size 0.2, will remain consistent and reliable at any warming stage of the i-CAT CBCT machine. The result of the first objective is a positive outcome for clinicians as they can remain confident of the high accuracy (within 0.007 mm) of the different machine warming stages of the CBCT scanning technology tested.

In terms of the second objective, the study showed that there was a statistically significant difference in average deviation between CT scanning with 1 mm slice thickness and CBCT scanning with 0.2 mm slice thickness. CT scanning with 1 mm slice thickness resulted in an average deviation from the control of 0.056 mm, and was shown to be less accurate than the CBCT scanning with 0.2 mm slice thickness at all machine warming stages where an average deviation from the control of 0.007 mm was found. Although there was a statistically significant difference between CBCT at 0.2 mm slice thickness and CT at 1 mm slice thickness, it appeared that there was no clinical significance to the result. The researcher discussed the results of the study with a head and neck surgeon and with a maxillofacial prosthodontist. Both discussions revealed that the results of the study were not considered clinically significant in the respondents’ practice of surgical design and preoperative planning. The head and neck surgeon revealed that any deviation in bone position under 1 mm would not be considered clinically significant in terms of osseous reconstruction, while the maxillofacial prosthodontist revealed that any deviation under 0.5 mm would not be considered clinically significant in terms of implant installation planning. While the relevance of dimension to different clinical specialists needs scientific definition, the differences cited are both well beyond the average deviation from the control of 0.056 mm for CT and 0.007 mm for CBCT. The result of the second objective is also a positive outcome for clinicians as CT medical imaging technology is a common means by which data are obtained from their patients. These findings are specific to the CBCT machine, materials and techniques used in the present study.

## Conclusion

The goal of the study on the accuracy of CBCT medical imaging technology was to assess whether there are differences between CT and CBCT in the form of 3D surface model reconstructions. For clinicians it is important to understand the parameters of the medical imaging technologies and what the differences are at different levels in order to obtain the optimal 3D surface model reconstruction.

## Abbreviations

CBCT: Cone beam computerized tomography; CT: Computerized tomography; 3D: Three-dimensional; CAD: Computer aided design; iRSM: Institute for reconstructive sciences in medicine; DICOM: Digital imaging and communications in medicine; DI: Diagnostic Imaging; .stl: Stereolithography

## Competing interests

The authors declare that they have no competing interests.

## Authors’ contributions

HL, JW, HS, BH and PB all conceived the study, participated in its design and coordination and drafted the manuscript. The present study was one of three studies done to complete a thesis for a Master of Science in Rehabilitation Science focusing in Surgical Design and Simulation (SDS). JW, HS, BH and PB all participated as supervisory committee members for the MSc of HL. All authors read and approved the final manuscript.

## Authors’ information

Heather Logan (HL) - MSc, BDes

HL is a Surgical Design Simulationist at the Institute for Reconstructive Sciences in Medicine (iRSM). She completed her Master of Science in Rehabilitation Science with a focus in Surgical Design and Simulation in 2011 at the University of Alberta. HL divides her time between clinical work in facial prosthetics, surgical design and simulation and research. Her research involves developing and refining surgical design methods and developing digital processes for facial prosthetic fabrication.

Dr. Johan Wolfaardt - BDS, MDent (Prosthodontics), PhD

JW is a Director of the Institute of Reconstructive Sciences in Medicine (iRSM) and is appointed as a Full Professor in the Faculty of Medicine and Dentistry, University of Alberta, Canada. His clinical and research interests are in the area of maxillofacial prosthetics with particular emphasis in the area of head and neck reconstruction, osseointegration and treatment outcomes. JW has led the development of the research program at COMPRU. His research interests involve treatment outcomes, digital technologies in head and neck reconstruction and biomechanics of osseointegrated implants. JW has published over 80 papers in refereed journals and contributed to a variety of texts. He has lectured both nationally and internationally on maxillofacial prosthetics, osseointegration in head and neck reconstruction, challenges of introduction of advanced digital technology, knowledge work, teamwork and quality management. JW is elected to the Boards of the International Society of Maxillofacial Rehabilitation, the American Academy of Maxillofacial Prosthetics and the International College of Prosthodontists.

Dr. Hadi Seikaly - MD, FR CSC

HS is a Professor of Surgery in the Department of Surgery (University of Alberta), Director of the Division of Otolaryngology Head and Neck Surgery, and the Zone Section Head for Otolaryngology Head and Neck Surgery. In addition, he is presently the president of the University Hospital Medical Staff Society, serves on several local, national, and international administrative committees and is on the Executive Council of the Canadian Society of Otolaryngology. HS graduated from the University of Toronto medical school and completed his residency training at the University of Alberta in Otolaryngology Head and Neck Surgery. He then obtained fellowship training at the University of Texas Medical Branch in advanced head & neck oncology, microvascular reconstruction and facial cosmetic surgery. Dr. Seikaly returned to the University of Alberta as an attending in the division of Otolaryngology, department of surgery in 1996 where he has been active in teaching, patient care, and research.

HS continues to have a large practice dedicated to head, neck and skull base oncology and reconstruction. His research interests include functional surgical and reconstructive outcomes, microvascular head and neck reconstruction, submandibular gland transfer and medical modeling as it applies to the head and neck region.

He has published more than 50 papers in peer reviewed journals and numerous chapters.

Dr. Pierre Boulanger – Ph.D., P.Eng

PB worked for 18 years at the National Research Council of Canada as a senior research officer where his primary research interests were in 3D computer vision, rapid product development, and virtualized reality systems. He now holds a double appointment as a professor at the University of Alberta in the Department of Computing Science and in the Department of Radiology and Diagnostic Imaging (Faculty of Medicine). PB is the Director of the Advanced Man Machine Interface Laboratory as well as the scientific director of the Alberta Radiological Visualization Center. His main research topics and teachings are on virtualized reality systems and medical imaging. He is also a Principal Investigator for Stereo IPTV at TRLabs. PB has published more than 260 scientific papers in various journals and conferences. PB is on the editorial board of two major academic journals as well as on many international committees and frequently gives lectures on rapid product development and virtualized reality. On the commercial side, PB is the President of PROTEUS Consulting Inc., an Alberta-based consulting firm specialized in Virtual Reality Applications.

Dr. Bill Hodgetts – Ph.D.

BH obtained his B.A. in Psychology and his M.Sc. in Audiology at the University of Western Ontario. He received his Ph.D. in Rehabilitation Sciences at the University of Alberta. BH is an Associate Professor in the Department of Speech Pathology and Audiology at the University of Alberta where he teaches in the areas of hearing science/audiology and research methods and statistics. He has a joint appointment with the Institute for Reconstructive Sciences in Medicine (iRSM), where he is program director of Bone Conduction Amplification. BH’s research involves developing and refining the selection, verification, and validation of fitting procedures for BAHA (bone anchored hearing aid). He also has a research interest in bone anchored hearing aids and noise exposure from MP3 Players.
